# Pathological Irregularities

**Published:** 1904-09

**Authors:** M. H. Fletcher

**Affiliations:** Cincinnati, Ohio


### PATHOLOGICAL IRREGULARITIES.[1]

BY M. H. FLETCHER, D.D.S., M.D., CINCINNATI, OHIO.

### ENUNCIATION.

THE terms orthodontia and irregularities of the teeth conventionally carry with them the idea of irregular teeth in children and youth, connected with their treatment for correction. The causes are usually hereditary, but may be acquired. One could quote from writers from Etruscan days down to the present time and give the opinion of more than fifty authors, but their definitions of the etiology would most likely each differ somewhat from the other.

There have been handed down to us such explanations as " She inherited large teeth from one parent and small jaws from the other," or " His baby teeth were not extracted soon enough," or " were taken out too soon." " Lack of absorption of the roots of the temporary teeth, while the growth of the permanent set is rapid," etc. One author thinks " the development of the hind end of the jaw does not keep pace with the absorption of the front end."

Then there are a lot of platitudes, such as " The teeth are too large for the jaw," " Too many teeth for size of the jaws," " Projecting jaws," " Sleeping with the mouth open," " Enlarged tonsils," " Want of room in the jaws," etc., etc.

In summarizing the above opinions it would seem that symptoms, or results, have been given in place of the real cause. Nevertheless, this is only another opinion.

### ETIOLOGY.

Guilford divides the causes into hereditary and acquired, and Colyer into general and local.

" Talbot has shown that irregularities of the teeth were often due to two factors. Those of constitutional origin, which develop with the osseous system, and those of local origin." " The deformity always commences at the sixth year and is completed at

[1] Read at the fifty-fifth annual session of the American Medical Association, in the Section on Stomatology, Atlantic City, June, 1904.

the twelfth." "Forward movement of the posterior teeth produce the same result as arrest of development of the maxillæ. It was also shown that the vault is not contracted by mouth-breathing. That contracted dental arches are as common among low as in high vaults and that they simply appear high because of the contraction. That mouth-breathing due to hypertrophy of the nasal bones and mucous membrane, deformities of the nasal bones, adenoids, or any pathologic condition producing stenosis, does not cause contracted jaws, but all these conditions are due to neuroses of development."

### EFFECTS.

The ill effects of these deformities must be apparent to such an audience as this with a mere suggestion. The degree and extent of the ill effects have not only to do with the unsightliness of the patient, but Talbot has done much to prove the connection of extreme cases with idiocy and crime.

Aside from uncomeliness, irregularities undoubtedly interfere with the proper care of the teeth and gums, and in this manner are a large factor in fostering diseases of the alveolar process, including the surrounding tissues; many times involving other parts of the jaws, the nose, eyes, and ears, often inducing chronic disorders of digestion and fostering the causes of zymotic diseases. Neuroses of many varieties may have their origin in diseased alveolar process and teeth.

### TREATMENT.

As to treatment, our best men differ in their procedures. Cleft palate and harelip are of course dealt with from a surgical standpoint. Prognathic cases, showing atavistic tendencies, with diastoma behind the canines, are sometimes treated surgically by removal of bone from these spaces, but such treatment is rare. In the treatment of lesser deformities mechanics are almost entirely relied on. Some operators resort to the removal of one or more teeth in order to accomplish the desired end. On the other hand, Dr. Angle says, "The best balance, the best harmony, the best proportions of the mouth in its relation to the other features, require in all cases that there shall be the full complement of teeth, and that each tooth shall be made to occupy its normal position. And if we accomplish this we shall have satisfied the demands of art, so far as they are concerned in the relation of the mouth to the rest of the face."

To restore the features to harmony and the teeth to perfect position and usefulness requires mechanical skill of the highest order, coupled with an æsthetic sense and artistic eye.

PATHOLOGIC IRREGULARITIES.

*Definition.*—In contradistinction to the above, there is a class of irregularities not treated of in works on orthodontia, nor have they been considered under the head of dental orthopædia. In fact, these cases seem in a way to be "the stones which the builders disallowed."

They are in many particulars the exact opposite of the others. 1. They do not appear until the age of mature years. 2. They are purely acquired. 3. They are entirely pathologic, in the sense that they are the result of disease, localized in the alveolar process. 4. They are only amenable to mechanical treatment by first removing the causes of the disease producing them.

*Name.*—In order to distinguish these from those previously described, the writer has called them pathologic irregularities.

*Etiology.*—To describe all the causes of pathologic irregularities would be to give a treatise on interstitial gingivitis, known also as pyorrhœa alveolaris and Riggs's disease.

To make the matter plain from my stand-point it will, however, be necessary to briefly describe the anatomy, the pathology, and the causes, with treatment other than mechanical.

*Anatomy.*—An intimate knowledge of the anatomy is of course necessary in order to comprehend the pathology, or to apply treatment intelligently. It is presumed this is understood.

Now, when we consider that a hard, unyielding substance like a tooth is not only supported and held in place by, but entirely dependent on, the thin, bony walls of the alveolar process, it is a marvel to realize what hard usage it withstands, and what enormous pressure and lateral strain it is continuously subjected to without displacement or injury. Let this bone become diseased, however, and ere long the teeth become tender and unusable, and vast numbers are finally lost without the least defect in the tooth itself.

In the last decade these diseases and their treatment have engaged the attention of the profession to a marked degree, much to its credit.

*Terminology.*—To Talbot is due the credit of having classified the various phases of this disease and described its different stages. He has given the name "interstitial gingivitis" to inflammation of the gums, alveolar process, and peridental membrane. The term Riggs's disease and pyorrhœa alveolaris were formerly applied to any or all the stages and conditions.

The term Riggs's disease is indefinite and is to-day obsolete. Pyorrhœa alveolaris now indicates a flow of pus from the sockets about the roots of the tooth, and is a terminal stage of inflammatory action. It is the result of previous inflammation known as interstitial gingivitis. Inflammatory action may continue, however, and exfoliation of the teeth result without pus infection. One termination of the inflammatory action is the tendency of the teeth to be expelled from their sockets, with the result that they become elongated, tilted to one side, or pushed in or out of the normal arch.

To give a plan of arresting this process before it has gone too far and to replace the teeth into their normal position is the object of this paper.

*Causes.*—In order to arrest or eradicate a disease, its causes must first be found and removed. Talbot says, "The local causes which produce interstitial gingivitis are an accumulation of tartar about the necks of the teeth, decayed teeth producing hypertrophy of the gums, unfinished fillings, gold crowns and bridge-work, artificial dentures, rapid wedging of the teeth, collections of food and everything that will produce irritation of the gum margin, setting up a chronic inflammation or gingivitis. This in turn extends to the deeper tissues (the peridental membrane and alveolar process), where it becomes interstitial in character. The constitutional causes which act locally, producing interstitial gingivitis, are the toxic effects of mercury, lead, brass, uric and other acids, potassium iodide, and other agencies acting in a similar manner, such as scurvy," etc.

He further says, "Autointoxication (meaning self-poisoning due to a faulty metabolism) is the great cause of interstitial gingivitis resulting in pyorrhœa alveolaris."

In contradistinction to investigators who hold that the disease is often entirely systemic, the writer's opinion is that the disease must have a local cause, this cause producing a point of least resistance for the localization of systemic disorders, which general disorder or condition of autointoxication increases the local symptoms.

There seems no reason to believe that drug poisoning or other morbid systemic conditions can produce interstitial gingivitis unless a lesion of the gum pre-exists. This lesion may be the merest break in the mucous membrane, caused by the smallest deposit of calcareous material, this local mechanical irritation being one requisite of the etiologic moment. On the other hand, there may frequently be found in gingivitis the systemic disorders accompanying cases of sapræmia and septicæmia.

The continual pressure against the gum tisssue of rough, irritating calcareous deposits, which continuously increase in quantity and insinuate themselves deeper and deeper beneath the soft tissues, are accompanied with all the products of repair by granulation or second intention, and may be accompanied by surgical fever. These deposits may be found wherever saliva can penetrate. It has never been my privilege to see deposits of tartar about the necks of teeth that were innoxious, but they are always irritating to some degree, and usually greatly so. This condition may exist in all stages, from that of being imperceptible to the naked eye up to a complete state of pyæmia, and may result in death.

On the other hand, there is abundant evidence to show that autointoxication, or a low state of health from any cause, greatly favors the progress of the disease, and with this state of affairs present a chronic pus-forming condition may soon be found about one or more of the teeth where the local exciting cause exists, but that autointoxication or other systemic disorders cause this disease, without local irritation, does not appeal to the writer's reason any more than to say that the same disorders cause inflammation of the pleura or conjunctiva without a local point of least resistance from local cause.

Degeneracy or faulty development may bring the etiologic moment at a very early stage of the local irritation. This might be almost coincident with the initial lesion, whereas in normal and healthy individuals the pyorrhœal stage, even in its mildest form, may be deferred indefinitely or never appear even where calcareous deposits are excessive.

The fact that the tissues involved are transitory in nature does not seem an adequate factor in accounting for the disease, as suggested by Talbot, since they are as transitory in cases where the disease does not exist as where it does, and these tissues recover as readily as other structures which are not transitory.

There seems no question but that calcareous deposits about the teeth should be looked on as noxious foreign bodies, and that the constant effort on the part of nature to extrude them results in the progressive death of the surrounding tissues, with the malposition of the teeth as one result. We find in this disease zones of granulation tissue with the result of destructive metabolism in the soft tissues and the creation of sequestra in the bone. This condition, however, is changed to constructive metabolism the moment the tartar, sequestra, or other local irritants are removed.

The sinus in the pyorrhœal stage of this disease is between the root and alveolar process, unless the lesion be so deep in some place on the outside of the process that a gingival abscess is formed. In either event the alveolar process is continually bathed in pus, which results in its destruction. So long as the tartar is present as a foreign body the irritation is continuous and sequestra are formed which are a second source of irritation until they are removed or absorbed.

All these cases will heal by removal of the deposits and sequestra or by the loss of the affected teeth. The removal of the teeth invariably results in recovery, and a patient without teeth, either young or old, cannot have the disease, regardless of transitory structures, degeneracy, heredity, drugs, environment, or systemic disease. If lesions of the gums or maxillary bones appear where there are no teeth, it is not interstitial gingivitis, but something else.

Of all the causes mentioned, the writer believes that ninety per cent. of cases of interstitial gingivitis are due to hard deposits about the teeth.

*Treatment.*—As to treatment, I believe that all authorities are agreed that absolute removal of all deposits about the necks and roots of teeth is the first requisite to recovery. In my own hands this requires from three to ten sittings, approximately a week apart, washing out the socket each time with hypodermic syringe, using fifty per cent. alcohol, saturated with boracic acid, painting the gums with iodine or iodide of zinc. They must then have constant care thereafter from one to six times a year in order to preserve a good state of health, or a "healthy stump," as surgeons say. Dr. W. A. Price, of Cleveland, has had good results by local treatment with the X-ray after having removed the deposits.

As to instruments, each one capable of doing the work will adopt his own methods and choose his own instruments and remedies for local treatment. If the diagnosis has been correctly made the practitioner will be the judge as to whether systemic interference be necessary. If constitutional treatment is called for, abstinence from excess of nitrogenous and acid foods, with the necessity of ten to twelve glasses of pure water daily and the addition of lithia for a period is usually indicated.

Much can be learned about the condition of the system by examination and analysis of the saliva and urine; neither should be more than slightly acid and both should be normal in other particulars.

Talbot says, " In the severer types of disease, such as tuberculosis, asthma, chronic indigestion, kidney disease, etc., very little curative effect is to be expected from treatment. Constitutional treatment is tentative, since autointoxication will continue in most cases until death. The chief treatment of such cases will be removal of local irritation.

" The system excretes forty ounces of water daily. If this amount be not taken into the system, or if it be not eliminated every twenty-four hours, autointoxication will follow. Every drop of water taken into the stomach enters the blood. It is one of the best purifiers which we possess. From five to seven pints of pure water should be taken each day to flush the blood and kidneys and thus cleanse the system."

*Mechanical Treatment.*—The causes having been determined and treatment carried well along, the malposition of the teeth should begin to have attention. This is usually begun before healing of the tissues is complete. The writer has had the most satisfactory results in these cases by straightening out their defects in the same manner that ordinary irregular teeth are treated. A description of the mechanical devices contrived and used for the purpose of regulating teeth would fill large volumes; yet in addition to all these the inventive powers of the operator are continuously called on in carrying these cases to satisfactory completion. In my own hands cumbersome regulating appliances have largely given way to a most simple plan,—namely, that of a simple bow of heavy German silver wire on the outside of the dental arch, so adjusted that the teeth are drawn to it by the use of ligatures of German silver or platinum instead of silk or rubber. Torsion is produced by putting on a band to which a tube is soldered; in this tube is inserted a spring lever, the outer end of which is ligated to the bow. The use of the bow on the outside of the arch is one of the oldest devices known, but the manner of its handling is varied, being susceptible of a great variety of uses.

The resiliency of the heavy bow is such that its steady pull or push moves the teeth out or pushes them into line. Its resiliency can also be utilized to expand or contract the strongest arch. It has nearly done away with jack-screws, Coffin plates, and many other intricate and annoying appliances where they were formerly used, and simplifies the treatment to a very great degree, and has done so in my hands for the past ten or more years.

This bow and its accessory appliances being entirely on the outside of the arch are much less annoying than appliances inside, and are very much more effective. It will be found that pathologic irregularities yield to pressure more readily than in younger persons because of the partial loss of alveolar process; then there are no short, partly erupted teeth to be dealt with.

Regarding the imaginary difficulty of changing the shape of bones in mature adults, it may be said that live bone never becomes so old that it will not yield to continuous pressure, and teeth are more easily replaced into a former position than moved into a new one. Nevertheless, two of these cases here presented show where adjoining teeth have been brought together and occupy spaces where a tooth had been extracted or lost from disease, both in patients fifty years of age.

As to changing the shape of bones, Dr. M. H. Cryer says, "After the birth of the child muscular action and various forces have direct influence over the change of the bones, according to the following general laws: The normal application of forces in developing bone results in the normal development of the form of the bone. The abnormal application of forces under the same circumstances results in the development of an abnormal form. Abnormal applications of forces to bone in adult life will also change and modify the shape and character."

These pathologic cases, like the others, must be retained in their new position for a period of months, may be years, or until the bony arch has become thoroughly ossified again. This is usually done by ligating them with platinum ligature. Sometimes a heavier platinum wire is fitted to the lingual surfaces and ligated to the teeth with the light platinum. The German silver and platinum ligature is No. 25 B. & S. gauge.

In November, 1893, I presented one of these cases, giving this plan of treatment, and read a paper on the subject before the Cincinnati Odontological Society. Since that time I have treated several additional cases with most satisfactory results.

DISCUSSION.

*Dr. Eugene S. Talbot,* Chicago.—I appreciate highly the new term coined by Dr. Fletcher, "pathologic irregularities" of the teeth. It is an important and common condition, and classified under this head defines the pathologic state. The tooth itself is to a great extent a foreign body in its relation to the alveolar process. The teeth, from want of antagonism, constantly move in the alveolar process due to interstitial gingivitis. This is particularly true of the old method of separating teeth by the rapid process for filling. An interstitial gingivitis was set up, because of this the teeth separated, and in later years a space resulted between the teeth. Because of the transitory nature of the alveolar process, an interstitial gingivitis always occurs after the second teeth have obtained their position. There is what may be called an inflammatory process continually going on in the alveolar process. This is the reason why a dental arch which has lost one or two teeth is always more or less out of order. This "inflammatory process" starts an absorption of the alveolar process, because of which the teeth move in different directions. How far such an alveolar process can be restored is an open question. Some operations of Dr. Fletcher, beautifully performed, bring the teeth back into place and hold them in position until the alveolar process is restored to partial health. It is never restored to complete health. This last is a physical impossibility. Local treatment is all right, so far as it goes. It is very essential that the deposits should be removed; that the roots of the teeth should be thoroughly cleansed, but besides that there is considerable to be done in regard to draining the system. It is necessary to restore the excretory organs to their function. Autointoxication is the great determining factor, no matter what the systemic condition may be. The greatest cause of this is intestinal fermentation.

